# Laparoscopic training on virtual-reality simulators or live pigs—a randomized controlled trial

**DOI:** 10.1097/MS9.0000000000000798

**Published:** 2023-06-05

**Authors:** Zhengqian Bian, Yan Zhang, Guangyao Ye, Feng Guo, Yifei Mu, Yinghui Fan, Xiang Zhou, Qing Zheng, Lars Konge, Zheng Wang

**Affiliations:** a Renji Hospital, Shanghai Jiaotong University School of Medicine, Shanghai; b Guangdong Academy for Medical Simulation (GAMS), Guangzhou, Guangdong, China; c Copenhagen Academy for Medical Education and Simulation (CAMES), Copenhagen, Denmark

**Keywords:** laparoscopic surgery, live animal surgery, simulation-based training, virtual-reality simulators

## Abstract

This randomized controlled trial compared the efficacy of virtual-reality (VR) simulator training and surgical training on live pigs to explore the most effective and evidence-based training modality.

**Materials and methods:**

Thirty-six novice surgical residents without independent laparoscopic experience were randomly paired with a peer and randomized into three groups: VR simulator group (dyad training on LapSim VR simulators), pig surgery group (training on live, anesthetized pigs) and control group (training by a lecture on laparoscopic surgery, surgical videos and textbooks). After 6 h of training, all participants performed a simulated cholecystectomy procedure using a pig liver with adherent gallbladder working in pairs. All procedures were video-recorded and the recordings were saved on USB-sticks in a blinded fashion identifiable only by the unique participant number. All video-recordings were scored blindly and independently by two expert raters using the Global Operative Assessment of Laparoscopic Skills (GOALS) assessment instrument.

**Results:**

The performances in the three groups were significantly different, *P* less than 0.001. Both the VR simulation training group and the live pigs training group performed significantly better than the control group, both *P* values less than 0.001. However, there was no significant difference in the performance of the two simulation-based training groups, *P*=0.66.

**Conclusion:**

Novice surgical trainees can benefit from both VR simulator training and pig surgery simulation compared with traditional studying and there was no significant difference between the two modalities. The authors recommend that VR simulators should be used for basic training of laparoscopic skills and surgery on live animals should be reserved for higher-level surgical training.

## Introduction

Highlights

- Hands-on training on both virtual-reality (VR) simulators and live pigs is more effective than traditional lectures and surgical videos.
- No statistical difference in learning efficacy was found between VR training and live animal surgery.
- VR simulators could replace live animals in basic training of laparoscopic skills.

Laparoscopic minimally invasive surgery has developed over the last decades resulting in smaller incisions, reduced postoperative pain, and shorter recovery times.^1–3^ However, laparoscopic surgery is more difficult to learn than open surgery and has a longer and more shallow learning curve.^4^ In the classic apprenticeship model, young surgeons conduct supervised training directly on patients^5^ causing longer operation times and more bleeding in the beginning of a novice surgeon’s learning curve.^6^

Simulation training allows for repeated practice at varying levels of difficulty in a more flexible environment without any risk to the patients. Several simulation-based teaching methods can be used in the basic training of laparoscopy, including virtual-reality (VR) simulators and operations on live, anaesthetized animals, for example pigs.^7^ Training on VR simulators avoids the ethical concerns associated with practice on animals or cadavers as well as exposure to blood-borne diseases. Furthermore, VR simulators offer automated metrics that can be used to provide immediate feedback to trainees (i.e. formative assessment) and decide when basic competency has been acquired (i.e. summative assessment).^8–10^ However, VR training also has disadvantages. Issues such as prohibitive cost of simulators, limited fidelity, poor familiarity of the techniques and surgical equipment can cause reluctance of faculty to allow time for its use and thereby prevent implementation of VR simulation into the surgical curriculum.^11,12^ Simulation-based training on live animals is closer to real surgery, offering familiarization to real surgical instruments, relatively similar anatomy, and the look, feel, and “reactions” (e.g. bleeding) of live tissue. However, live animal surgery requires special facilities and dedicated personnel to care for the animals and provide instructions and feedback to the trainees which make repeated training sessions logistically challenging and expensive.^13^

Optimally, surgical educators should use the most effective and evidence-based training modality but there is a lack of research on this topic. The aim of this study was to explore and compare the efficacy of VR simulator training and surgical training on live pigs.

## Methods

This randomized controlled trial was conducted at the simulation centre at the Renji Hospital, Shanghai, China where dedicated facilities exist for both VR simulator training and surgery on live pigs. The study was approved by the local institutional review board/ethics committee on the 23 of December 2020, protocol no. RJ-2020007-2 and results were reported according to the CONSORT guidelines using the extension for health care simulation research.^14^ The work was registered with the Research Registry with the unique identifying number: researchregistry8758. https://www.researchregistry.com/browse-the-registry#home/registrationdetails/640eee407e6bc100287b4dae/.

**FIGURE 1:**
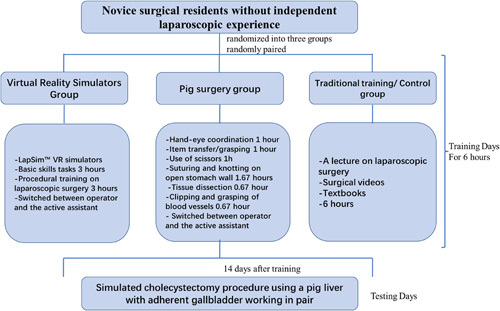
The Global Operative Assessment of Laparoscopic Skills (GOALS) assessment tool. Depth perception: 1—Constantly overshoots target, wide swings, slow to correct. 3—Some overshooting or missing target, but quick to correct. 5—Accurately directs instruments in the correct plane to target. Bimanual dexterity: 1—Uses only one hand, ignores non dominant hand, poor coordination between hands. 3—Users both hands, but does not optimize interaction between hands. 5—Expertly uses both hands in a complementary manner to provide optimal exposure. Efficiency: 1—Uncertain, inefficient efforts; many tentative movements; constantly changing focus or persisting without progress. 3—Slow, but planned movements are reasonably organized. 5—Confident, efficient and safe conduct, maintains focus on task until it is better performed by way of an alternative approach. Tissue handling: 1—Rough movements, tears tissue, injures adjacent structures, poor grasper control, grasper frequently slips. 3—Handles tissue reasonably well, minor trauma to adjacent tissue (i.e. occasional unnecessary bleeding or slipping of the grasper). 5—Handles tissues well, applies appropriate traction, negligible injury to adjacent structures. VR, virtual-reality.

Participants were novice surgical residents without independent laparoscopic experience. They were divided into three classes of 12 and came to the simulation centre where they received verbal and written information about the study and signed informed consent. Each participant got a unique identification number (First class: 1–12, second class 13–24, and third class 25–36). Then they were randomly paired with a peer and randomized into three groups: VR simulator group, pig surgery group, and control group according to a balanced randomization key generated using www.random.org. Immediately following the randomized pairing, each pair signed up for at “testing slot” (please see below) and then started training according to their group. Figure 1 shows an overview of the study design.

### Training days (1 day per class—3 in total)

The two pairs in the VR simulator group immediately started dyad training on two LapSim VR simulators (Surgical Science) following a standardized training program consisting of a total of 6 h of training where the participants switched roles between being the operator and the active assistant holding the camera. The program was a revised version of the training described in earlier publications.^15,16^ Initially, all participants familiarized themselves to the simulator by practicing six basic skills tasks for isolated skills that is coordination, instrument navigation, grasping, lifting and grasping, fine dissection, and cutting. After each attempt, both participants studied the automated feedback consisting of different metrics provided by the simulator, for example time, instrument path length, misses, collisions, damages, etc. After ~3 h of basic skills training, the participants proceeded to procedural training on two different procedures, a laparoscopic appendectomy and a laparoscopic salpingectomy due to an ectopic pregnancy. The training was supervised by an instructor who ensured that all four trainees received approximately the same amount of hands-on training and provided feedback to the trainees.

The two pairs in the pig surgery group transferred to the live animal facilities where they practiced on two live, anesthetized pigs. During 6 h of training, the participants switched roles between operator and assistant resulting in 3 h of hands-on surgery and 3 h of camera navigation training for both. Training followed a standardized setup including exercises focusing on hand-eye coordination (60 min), item transfer/grasping of omemtum tissue (60 min), use of scissors on bladder wall/abdominal wall (60 min), suturing and knotting on open stomach wall (100 min), tissue dissection of blood vessels around the stomach and small intestine (40 min), clipping and grasping of blood vessels (40 min). One experienced surgeon supervised the training, provided feedback, and balanced the roles of operator and assistant for both pairs of trainees.

The two pairs in the control group went to a separate room in the simulation centre where they received a lecture on laparoscopic surgery and were provided access to surgical videos and textbooks. They were urged to spend 6 h in total studying the available material.

### Testing days

Each pair returned to the simulation centre for testing according to the model initially described by Brinkmann *et al*.^17^ The model consists of a pig liver with adherent gallbladder that is placed in a tray with gallbladder on top and the cystic duct and artery located medially. The participant with the lowest number started as operator assisted by his/her “buddy”. After completion of the first simulated cholecystectomy procedure, the participants had a five-minute break before switching places and repeating the procedure with a new specimen. All procedures were video-recorded and the recordings were saved on USB-sticks in a blinded fashion identifiable only by the unique participant number. All testing slots were scheduled 14 days after the training day.

### Scoring

All video-recordings were scored blindly and independently by two expert raters using the Global Operative Assessment of Laparoscopic Skills (GOALS) assessment instrument (Figure 1). The instrument includes four items that are scored from 1 to 5 points. All scores were recoded into 0–4 points as in the original Objective Structured Assessment of Technical Skills (OSATS)^18^ resulting in a possible total score ranging from 0 to 16 points.

### Statistical analysis

The internal consistency reliability of the GOALS was calculated as Cronbach’s alpha and the inter-rater reliability was ensured by calculating Pearson’s r. A mixed model was applied to assess differences between intervention groups. Intervention type was treated as fixed effect and raters were treated as repeated measurements to account for the correlation between the rater scores for each participant. A compound symmetry covariance structure was specified for the intra-rater variance, thus assuming same variance for the two raters and a single covariance measure. Post hoc comparisons were performed using Bonferroni correction of *P* values. The analysis was performed with the MIXED procedure from the software package IBM Statistical Package for the Social Sciences (SPSS) version 25. *P* values below 0.05 were considered statistically significant.

## Results

There were no statistically significant differences between the 3 groups in demographic factors as shown in Table 1.

**TABLE 1:** Demographic data of participants.

	Virtual-reality Simulators	Live, anesthetized animals	Traditional training (control group)
Age (mean±SD)	21.5±0.8	21.4±0.7	21.8±0.6
Sex ratio (F:M)	6:6	6:6	6:6
Semester	7	7	7
Handedness (right:left)	12:0	12:0	12:0
Training time	6 h	6 h	6 h
Experience in laparoscopic surgery	None	None	None

F, female; M, male.

The internal consistency reliability of the GOALS assessment tool was very high, Cronbach’s alpha = 0.91 and the inter-rater reliability was high, Pearson’s r = 0.81.

Table 2 shows the scores of the three randomized groups. The performances in the three groups were significantly different, *P* < 0.001. The VR simulation training group performed significantly better than the group using traditional training methods (lecture, books, and videos), mean GOALS scores 8.96 versus 4.63, *P* less than 0.001. Participants training on live pigs were also significantly better than traditionally trained participants, mean GOALS scores 9.33 versus 4.63, *P*<0.001. However, there was no significant difference in the performance of the two simulation-based training groups, difference in mean GOALS scores = 0.37, *P*=0.66.

**TABLE 2:** The mean GOALS scores and the 95% CI of the three randomized groups.

Group	Mean GOALS score (0–16) (95% CI)	Depth perception (0–4) (SD)	Bimanual dexterity (0–4) (SD)	Efficiency (0–4) (SD)	Tissue handling (0–4) (SD)
VR simulator training (*n*=12)	8.96 (7.74–10.18)	2.33 (0.65)	2.46 (0.54)	2.38 (0.43)	1.79 (0.94)
Comparison VR versus pigs	*P*=0.70	*P*=0.43	*P*=0.86	*P*=0.84	*P*=0.65
Training on live pigs (*n*=12)	9.33 (8.16–10.55)	2.54 (0.62)	2.50 (0.56)	2.33 (0.58)	1.96 (0.81)
Comparison pigs versus traditional	*P*<0.001	*P*<0.001	*P*<0.001	*P*<0.001	*P*<0.001
Traditional training using lecture, books, and videos (control group) (*n*=12)	4.63 (3.40–5.84)	1.21 (0.45)	1.50 (0.43)	1.33 (0.44)	0.58 (0.42)

GOALS, Global Operative Assessment of Laparoscopic Skills; VR, virtual-reality.

Scores from the four sub-themes of the GOALS assessment (depth perception, bimanual dexterity, efficiency, and tissue handling) showed that the participants in all three groups struggled especially with tissue handling where they achieved their lowest mean scores.

## Discussion

Our randomized controlled trial compared three different training modalities for laparoscopic surgery and found significantly different performances in the three groups, *P* less than 0.001. Post hoc analysis revealed that the reason for this difference was the superior performance of both simulation groups (training on VR simulators and live pigs, respectively) compared with the control group that had traditional lectures and watched surgical videos. This finding is not new as several previous studies have proven the efficacy of both VR simulation training and pig surgery.^19–21^ Our aim-of-study and main finding was the direct comparison of VR simulation and training on live, anesthetized animals that showed no significant difference in the performance of these two simulation training groups, *P*=0.66. The high and not significantly different efficacy of these training modalities means that surgical educators are free to use both when planning simulation-based training programs for novice surgical trainees in their own local context. However, there are still several factors that need to be considered such as realism, possibility for feedback/certification, costs, and ethics. Table 3 shows a brief overview of these.

**TABLE 3:** Perceived pros and cons of simulation-based laparoscopy training using VR simulators and live, anesthetized animals (LA), respectively.

	VR Simulators	LA	Item favors
Anatomical realism	++	+	VR
Tissue realism	-	++	LA
Teach novices correct use of surgical tools	+	++	LA
Opportunity to practice surgical emergencies	0	++	LA
Prepare novices for real operations	++	++	Both: equal effect according to current study
Prepare experienced surgeons for advanced procedures	+	+	Unknown, but LA is probably best
Easy access to feedback during training (formative assessment)	++	-	VR
Possibility for mastery learning (i.e. continued training to pre-defined level of proficiency – summative assessment)	++	-	VR
Resources needed to initiate training program	--	--	Neither—both training modalities are expensive to start
Resources needed to run training program	+	-	VR
Ethical concerns regarding animal rights	++	--	VR

-- = great disadvantage, - = disadvantage, 0 = neutral, + = advantage, ++ = great advantage.

LA, live, anesthetized animals; VR, virtual-reality.

Intuitively, a surgical simulation should resemble a real operation to avoid a “transfer gap” when the trainee moves from the simulation centre to the real operating room.^22^ The human and animal anatomy are quite similar, but differences do exist that might cause problems when novice, simulation-trained surgeons start operating on patients. The specific procedural modules of the VR simulators have software that mimics real human anatomy but the look and feel of the simulated human tissue is still not completely life-like despite delicate graphics and advanced force-feedback hardware. This is much better in live animals where the trainees can also get used to the real surgical instruments and not “only” simulated forceps, staplers etc. Another advantage of live animals is the possibility to simulate surgical disasters, for example major bleedings, that could become very useful if the trainee encounter a similar situation during real surgery. The VR software also includes some of these emergency cases but the realism and usefulness of these remain to be proven in scientific studies.^23^ These considerations and the results of the current study indicate that novice trainees should acquire basic laparoscopic skills on a VR simulator and then proceed to practice on live animals to get familiar with real tissue, real surgical instruments, and surgical emergencies.^24,25^ The rationale beyond this stepwise approach is also supported by studies showing increased cognitive load leading to decreased learning when complete novices start training in too advanced (i.e. too realistic) environments.^22,26^

It is important to acknowledge that simulation equipment is not the only determining factor for the learning outcome of simulation-based training. The design of the training program is also important and studies have shown beneficial effects of both access to guiding and supporting feedback during training (i.e. formative assessment)^27,28^ and of the mastery learning principle where an end-of-training test forces trainees to continue training until they can pass (i.e. summative assessment).^29^ The VR simulators deliver automatic performance metrics after each procedure that can be used for both formative and summative assessment whereas an experienced surgeon is necessary to give feedback to the trainees during surgical training on live animals. Setting up a mastery learning training program is relatively easy using VR simulators that allow trainees to conduct the amount of individual training lessons that is necessary for each trainee to pass the test. It is also possible and recommendable to perform a final test at the end of a pig training course, but this again requires an experienced supervisor and subjectivity and bias are difficult to avoid. Furthermore, according to the mastery learning principle an entire new course needs to be arranged for the trainees that fail the test which will increase the cost of simulation-based training considerably.

Cost and cost-effectiveness are major concerns that affect acceptability and implementation of surgical simulation training.^30,31^ According to Levin’s Framework for Cost Ingredients in Standard Educational Interventions, the costs contain facility costs, personnel costs, equipment and materials costs, client inputs, and other program inputs.^32^ Both VR simulator and pig surgery were much more expensive than the traditional didactic lecture and access to surgical textbooks and videos that we used for our control group. Surgical training on live animals includes the costs of building a special animal operating room, acquiring surgical instruments, hiring special operating staff, and buying animals.^33^ For VR training, the cost of investment is mainly in the purchase of VR simulators and maintenance, and require lower investment in venues, personnel, etc. So, surgery on live anaesthetized animals bears higher cost than VR simulators^34^ and should not be used to train basic laparoscopic skills from a cost-effectiveness point of view.

Reserving the use of live animals to more advanced surgical training also makes sense from an ethical point of view. Live animals should not be used for training when there are other training modalities with similar effectiveness as shown in our study. In some countries such as the United Kingdom, the use of live animals for surgical training is not allowed^35^ and here we must work even harder to find good alternative training methods and prove the efficacy of these.

The main strength of this study is the comparison between VR simulator training and live animal surgery simulation in a randomized setup using blinded raters. Most surgeons agree that simulation should be used to improve surgical training and patient safety, but a lot of even very basic skills training still takes place on patients. In a survey of 738 surgeons,^36^ 636 (86.2%) believed that laparoscopic simulation training should be emphasized but only 141 (19.1%) had received laparoscopic simulation-based training courses, and only 129 (17.5%) were required to prove their laparoscopic abilities in a formal test before operating on patients. In order to overcome this implementation gap, we need high quality medical education research exploring the most optimal simulation training modalities regarding trainees’ competence levels, feasibility, costs, and ethical concerns.

Our study has several limitations. Generally, studies in medical education have a low number of participants (on average *n*=30^37^) and our study was also relatively small (*n*=36). This introduces the risk of committing a type-II statistical error by overlooking a difference in performance of our two interventions groups. However, the mean scores of the participants in the animal training group were only 4% bigger than the VR simulator trainees and the clinical importance of a difference of this magnitude would be too small to change the study’s conclusions. The goal of simulation-based training is to achieve good transfer to the clinical world and training studies should strive to measure training efficacy in the real operating room. However, this would not be practically feasible in our setup where the heterogeneity of patients and especially the concerns for patient safety would make it very difficult to assess the real clinical skills of our novice trainees. Instead, we used blinded assessments of performances on an existing test with established evidence of validity. The good internal consistency reliability, good inter-rater reliability, and the very significant differences in performances across groups support the use of this test. However, the test mainly focusses on the technical skills of the trainees and it is important to acknowledge that non-technical skills also influence the patients’ safety and should be trained using simulation as well.^38,39^ Simulation-based training is superior to traditional didactic non-technical skills training courses^40,41^ and it is possible to train non-technical skills including decision making, situational awareness, managing stress, coping with fatigue etc. in a simulated setup. This could be done using either VR simulators or live animals but is outside the scope of the current study.

## Conclusions

Novice surgical trainees can benefit from both VR simulator training and pig surgery simulation compared to traditional studying and there was no significant difference between the two modalities. Based on considerations regarding training efficacy, need for supervisors, costs, and animals’ ethics, we recommend that VR simulators should be used for basic training of laparoscopic skills and surgery on live animals should be reserved for higher-level surgical training.

## Ethical statement

The authors are accountable for all aspects of the work in ensuring that questions related to the accuracy or integrity of any part of the work are appropriately investigated and resolved.

## Informed consent and patient details

All participants volunteered to participate and signed informed consent. No patients were involved in the study.

## Source of funding

This research did not receive any specific grant from funding agencies in the public, commercial, or not-for-profit sectors.

## Author contribution

Z.B.: conception and design of the study, acquisition of data, drafting the article, final approval of the version to be submitted. L.K.: conception and design of the study, analysis and interpretation of data, drafting the article, final approval of the version to be submitted. Y.Z., G.Y., F.G., Y.M., Y.F., X.Z., Q.Z., Z.W.: acquisition of data, critically revising the article for important intellectual content, final approval of the version to be submitted.

## Conflicts of interest disclosure

None.

## Research registration unique identifying number (UIN)

The work was registered with the Research Registry with the unique identifying number: researchregistry8758. https://www.researchregistry.com/browse-theregistry# home/registrationdetails/640eee407e6bc100287b4dae/.

## Guarantor

Professor Lars Konge, MD PHD, accept full responsibility for the work and the conduct of the study, had access to the data, and controlled the decision to publish.

## Data availability statement

The dataset generated and analyzed during the current study is available upon reasonable request.

## Provenance and peer review

Not commissioned, externally peer-reviewed.
